# Molecular Cloning and Expression Responses to *Streptococcus agalactiae* and *Aeromonas veronii* of TLR19, TLR20, and TLR21 in *Schizothorax prenanti*

**DOI:** 10.3390/ani16030511

**Published:** 2026-02-05

**Authors:** Qiyu Luo, Jie Zhang, Yao Shi, Yanjing Zhao, Yuanchao Zou, Xianghui Kong

**Affiliations:** 1 Henan Province Engineering Research Center of Aquatic Animal Disease Control, College of Fisheries, Henan Normal University, Xinxiang 453007, China; fionaksm@163.com (Q.L.); sysy2024123@163.com (Y.S.); yanjing217907@126.com (Y.Z.); 2 Conservation and Utilization of Fishes Resources in the Upper Reaches of the Yangtze River Key Laboratory of Sichuan Province, College of Life Sciences, Neijiang Normal University, Neijiang 641100, China; zou3891@163.com

**Keywords:** Toll-like receptors, *Schizothorax prenanti*, *Streptococcus agalactiae*, *Aeromonas veronii*

## Abstract

Toll-like receptors (TLRs) are essential pattern recognition receptors of the innate immune system and play critical roles in pathogen invasion in teleosts. In this study, we identified and characterized full-length open reading frames of three TLRs belonging to the TLR11 subfamily from *Schizothorax prenanti*, termed *spTLR19* (2868 bp), *spTLR20* (2835 bp), and *spTLR21* (2946 bp), encoding 955, 944, and 981 amino acids, respectively. All three proteins exhibited the conserved domain architecture typical of TLRs, comprising a leucine-rich repeat (LRR) domain, a transmembrane region, and a Toll/IL-1 receptor (TIR) domain. Phylogenetic and homology analyses revealed that *sp*TLR19 and *sp*TLR20 clustered most closely with their homologues from *Cyprinus carpio*, while *sp*TLR21 showed the highest similarity to *Onychostoma macrolepis* TLR21. Expression profiling showed that these TLRs were ubiquitously expressed across examined tissues, with relatively higher expression in immune-related tissues such as spleen and gills. Furthermore, challenge with *Streptococcus agalactiae* and *Aeromonas veronii* significantly up-regulated the expression of *spTLR19*, *spTLR20*, and *spTLR21* in spleen, liver, and gills, suggesting their involvement in antibacterial immune responses. These findings enhance the functional understanding of the teleost TLR11 subfamily and provide a foundation for elucidating disease resistance and immune regulation in *S. prenanti*.

## Simple Summary

As essential pattern recognition receptors of the innate immune system, Toll-like receptors (TLRs) are critical for pathogen recognition in teleosts. The transitional cold-water fish *Schizothorax prenanti*, distributed from low- to high-altitude regions, serves as an important species for elucidating immune mechanisms in cold-water fish and adaptations to environments with altitude variation. Three TLRs (TLR19, TLR20, TLR21) were identified from *S. prenanti*, each possessing the conserved domain architecture typical of TLRs and involved in antibacterial immunity. These findings enhance understanding of TLR functions in teleost innate immunity and provide a foundation for elucidating disease resistance and immune regulation in *S. prenanti*.

## 1. Introduction

In vertebrates, the immune system generally comprises innate and adaptive immunity. Teleosts, as lower vertebrates, depended more heavily on innate immunity for host defense compared to mammals [1]. Pattern recognition receptors (PRRs), as key components of the innate immune system, recognize pathogen-associated molecular patterns (PAMPs) and damage-associated molecular patterns (DAMPs), thereby initiating rapid and non-specific immune responses. The main PRRs identified in teleosts include Toll-like receptors (TLRs), NOD-like receptors (NLRs), RIG-I-like receptors (RLRs), and C-type lectin receptors (CLRs) [2,3,4]. As the first and best characterized PRRs, TLRs play an essential role in innate immunity and are conserved in invertebrates and vertebrates [5]. Structurally, TLRs characteristically contain an extracellular leucine-rich repeat (LRR) domain, a transmembrane region, and an intracellular Toll/interleukin-1 receptor (TIR) domain [6]. To date, at least 20 distinct TLRs have been identified in teleosts and are phylogenetically classified into six subfamilies: TLR1, TLR3, TLR4, TLR5, TLR7, and TLR11 [7].

TLR19, TLR20, and TLR21 belong to the TLR11 subfamily, the only one that does not contain any human TLRs, and research on this subfamily remains scarce [7,8]. Both TLR19 and TLR20 were fish-specific TLRs. TLR19 was first identified in zebrafish (*Danio rerio*) and has since been detected in other fish species such as barbel chub (*Squaliobarbus curriculus*), grass carp (*Ctenopharyngodon idella*), and *Gymnocypris przewalskii* [9,10,11]. In grass carp kidney cells (CIKs), the mRNA expression of TLR19 was up-regulated significantly by poly(I:C), but there was no response to LPS, PGN, and dsDNA [12]. In yellow catfish (*Pelteobagrus fulvidraco*), TLR19 was up-regulated in response to *Aeromonas hydrophila*, LPS, and PGN [13]. However, in blunt snout bream (*Megalobrama amblycephala*), TLR19 was down-regulated after *A. hydrophila* challenge [14]. TLR20 was also first identified in *D. rerio*, where multiple copies have been discovered [9]. In addition, expression of TLR20 in loach (*Misgurnus anguillicaudatus*) was significantly up-regulated after treatment with *A. hydrophila* [15]. In grass carp, three TLR20s (named CiTLR20.1 to CiTLR20.3) were identified and exhibited different responses to the same pathogen [16,17]. CiTLR20.2 and CiTLR20.3 could be induced by infection with grass carp reovirus (GCRV) and *A. hydrophila*. In contrast, CiTLR20.1 was insensitive to viral and bacterial infections [16]. Similar findings were reported in common carp (*Cyprinus carpio* L.), which exhibited two functional ferroportins with distinct expression patterns, suggesting potential functional divergence between them [18]. TLR21, a non-mammalian TLR, has been identified in several fish species, including largemouth bass (*Micropterus salmoides*) [19], mandarin fish (*Siniperca chuatsi*) [20], golden pompano (*Trachinotus ovatus*) [21], blunt snout bream (*M. amblycephala*) [22], grass carp (*C. idella*) [23], and so on. After *A. hydrophila* infection, TLR21 expression was significantly up-regulated in the liver and spleen of grass carp, while it was up-regulated in the liver but down-regulated in the spleen of blunt snout bream [22,23]. Additionally, in largemouth bass and golden pompano, TLR21 was induced by LPS and poly(I:C) stimulations in different tissues [19,21,24]. However, in head kidney lymphocytes (HKLs) of mandarin fish, the expression of TLR21 was down-regulated after LPS and poly(I:C) stimulation [20]. Therefore, expression patterns of one TLR in different fish species responding to the same pathogen or PAMP were distinct, implying that the function of TLR19, TLR20, and TLR21 may be species-specific. Unfortunately, these three TLRs in *Schizothorax prenanti* have not been reported, and their function in this species also remains unclear.

*Schizothorax prenanti*, commonly known as “Ya fish,” is a benthic cold water fish species belonging to the Cyprinidae, Schizothoracinae, and *Schizothorax*. Endemic to the upper reaches of the Yangtze River, primarily in the Jinsha River and Dadu River at an average altitude of around 1000 m, it is a regionally important economic species. However, its natural populations are declining rapidly due to a slow growth rate and habitat alterations from water conservancy projects. Consequently, artificial breeding has become essential for its conservation and cultivation. Intensive aquaculture, while a solution, increases the risk of pathogen transmission. *S. prenanti* is particularly susceptible to bacterial pathogens such as *A. hydrophila*, *A. veronii*, and *Streptococcus agalactiae* [25]. To date, studies on the immune responses of cold-water fish to pathogenic microbial infections remain limited. Given its status as a transitional cold-water species distributed from low to high-altitude regions, *S. prenanti* holds important significance for understanding the immune mechanisms in cold-water fish and adaptations to environments with altitude variation.

In this study, three TLR11 subfamily members (*TLR19*, *TLR20*, and *TLR21*) were identified and characterized from *S. prenanti*, designated as *SpTLR19*, *SpTLR20*, and *SpTLR21*, respectively. Their tissue distribution and transcription responses in different tissues following challenge with *S. agalactiae* and *A. veronii* were investigated. The aims of this work were to determine the structural features, expression patterns, and potential immune functions of these TLRs, and to elucidate the immune responses of *S. prenanti* to infection of *S. agalactiae* and *A. veronii*. The present study will further deepen our understanding of the immune functions of TLR19, TLR20, and TLR21 in antibacterial immunity and provide new insights for developing new strategies of disease control to protect the transitional cold-water species from pathogen infection.

## 2. Materials and Methods

### 2.1. Fish and Challenge

*S. prenanti* (10 ± 2 g) were obtained from Sichuan Ya Fish Company (Meishan, China) and bred in 60 × 40 × 50 cm^3^ tanks containing Chlorine-free freshwater under a natural photoperiod. After an adaptation period of two weeks, the fish were randomly divided into three groups (30 fish per group). For the treatment groups, the fish were injected intraperitoneally with 10 μL/g body weight of *S. agalactiae* (5.4 × 10^7^ CFU/mL) or *A. veronii* (6.3 × 10^7^ CFU/mL), respectively. In the control group, the fish were injected with the same volume of PBS. After treating 6, 12, 24, 48, and 72 h, the fish were euthanized using MS-222, then the spleen, head kidney, liver, and gills were collected from each fish, respectively, and stored in −80 °C until total RNA extraction. Three fish were sampled from each group at each time point, and the experiments were performed in triplicate.

### 2.2. RNA Extraction and cDNA Synthesis

Total RNA was, respectively, extracted from the collected different tissues using the RNA isolator Total RNA Extraction Reagent (Vazyme Biotech Co., Ltd., Nanjing, China) following the manufacturer’s instructions. The concentration and purity of the RNA were measured using a NanoDrop 2000c (Thermo Fisher Scientific Inc., Waltham, MA, USA). First-strand cDNA for gene cloning and quantitative Real-Time PCR (qRT-PCR) was synthesized using the HiScript II 1st Strand cDNA Synthesis Kit and the HiScript II Q RT SuperMix for qPCR (Vazyme Biotech Co., Ltd., Nanjing, China), respectively, following the manufacturer’s protocols. The obtained cDNA was stored at −20 °C until used.

### 2.3. CDS Cloning of spTLR19, spTLR20, and spTLR21

The primers were designed based on the conserved regions of known TLR sequences from *Schizothorax* species and other Cyprinids (Table S1) and synthesized by Sangon Biotech (Shanghai, China). Spleen cDNA was used as the template for amplification of *spTLR19*, *spTLR20*, and *spTLR21*. PCR amplification was conducted using the thermal cycler (Bio-Rad Laboratories, Hercules, CA, USA), and performed in conditions as follows: 94 °C for 4 min, then 34 cycles of 95 °C for 30 s, 53–56 °C (for different fragments of TLRs) for 30 s, and 72 °C for 2 min, followed by a final extension at 72 °C for 8 min. The PCR products were ligated with pMD19-T vector (Takara Bio Inc., Shiga, Japan), and then transformed into *Escherichia coli* DH5α competent cells (Vazyme Biotech Co., Ltd., Nanjing, China). After that, the competent cells were cultured on LB agar plates (containing 100 mg/L ampicillin) at 37 °C. Subsequently, positive clones were screened by colony PCR, and at least two clones were sequenced in both directions (Genewiz from Azenta Life Sciences, Suzhou, China). The newly deposited TLR19, TLR20, and TLR21 sequences were deposited in GenBank under accession numbers PX610149, PX610150, and PX610151, respectively.

### 2.4. Sequence Analysis, Phylogenetic Analysis, and 3D-Homology Modeling

The amplified sequences were compared with homologous sequences from closely related species using BLAST 2.17.0 on the NCBI (https://blast.ncbi.nlm.nih.gov/; accessed on 3 May 2024). Obtained sequences were identified using the BLASTn program and considered valid if they met the following thresholds: E-value = 0, percentage identity > 90%, and query coverage > 98%. The Neighbor-Joining phylogenetic tree was constructed based on the deduced amino acid sequences using the MEGA 11 program with 1000 bootstrap replicates. Additionally, the molecular weight and isoelectric point (pI) of *sp*TLRs were predicted using the ProtParam tool (https://web.expasy.org/protparam/; accessed on 12 July 2024). The domain architecture of the *sp*TLRs was identified according to the method described by Matsushima et al. [26]. Subsequently, the 3D structures were predicted using SWISS-MODEL (https://swissmodel.expasy.org/; accessed on 15 October 2024), and the models were further analyzed and refined with PyMOL 3.1.6.1 to visualize the protein structure and annotate secondary structure elements, such as α-helices and β-sheets.

### 2.5. Quantitative Real-Time PCR Assay

Total RNA was extracted from various tissues (head kidney, trunk kidney, brain, spleen, liver, gills, heart, muscle, intestine, and skin), and cDNA was prepared as described above. qRT-PCR was performed using ChamQ Blue Universal SYBR qPCR Master Mix (Vazyme Biotech Co., Ltd., Nanjing, China) on a Roche LightCycler 480 (Roche, Basel, Switzerland) in a 10 μL reaction volume. The primers were designed using Primer 5.0 software, and *18S rRNA* was used as the internal control (Table S1). The qRT-PCR conditions were as follows: initial denaturation at 95 °C for 2 min, followed by 40 cycles of 95 °C for 10 s, 60 °C for 20 s, and 72 °C for 15 s, with a final extension at 72 °C for 3 min. The prime amplification efficiency of *spTLR19*, *spTLR20*, and *spTLR21* were 98.55%, 97.98%, and 99.25%, respectively. The same method was also used to investigate the response of TLRs to bacterial challenge.

### 2.6. Statistical Analysis

The mRNA expression levels of the target genes relative to the reference gene (*18S rRNA*) were calculated using the 2^−ΔΔCt^ method [27]. The data were presented as mean ± standard error of the mean (SEM) (*n* = 3). Statistical analysis was performed by GraphPad Prism 9.3.1 (San Diego, CA, USA) and determined using one-way analysis of variance (ANOVA). The *p* value less than 0.05 was considered statistically significant.

## 3. Results

### 3.1. Identification of spTLR19, spTLR20, and spTLR21

**Figure 1:**
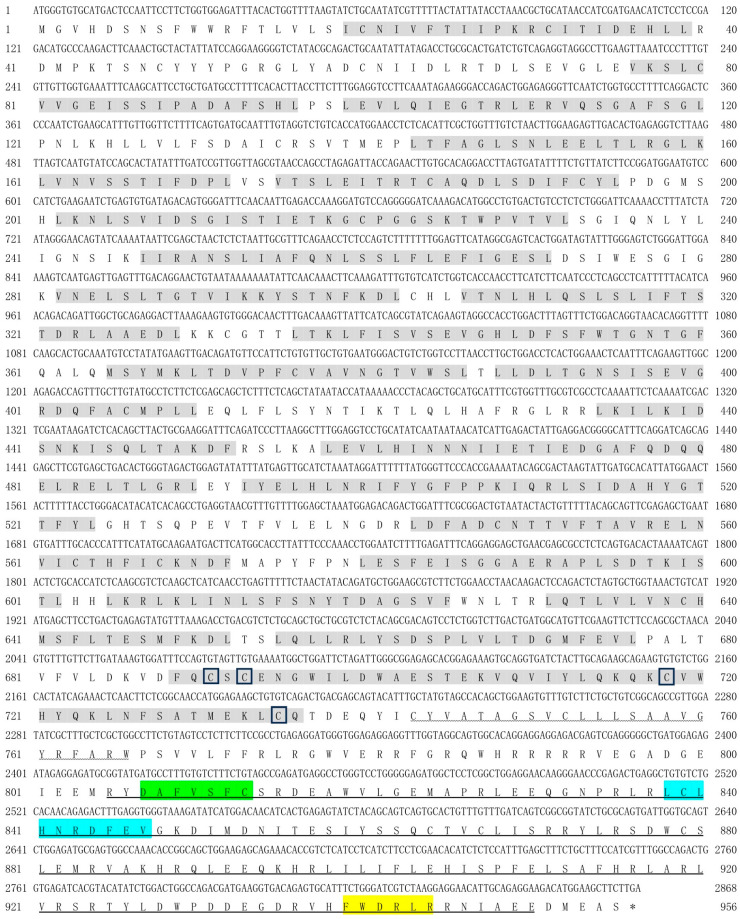
Nucleotide and deduced amino acid sequences and the domain architecture of spTLR19. The shaded regions indicate the LRR domains, the wavy line marks the transmembrane domain, and the horizontal line represents the TIR domain. The cysteine residues marked within the boxes indicate conserved cysteine sites. The green region represents Box 1, the blue region represents Box 2, and the yellow region represents Box 3. * represents the stop codon.

**Figure 2:**
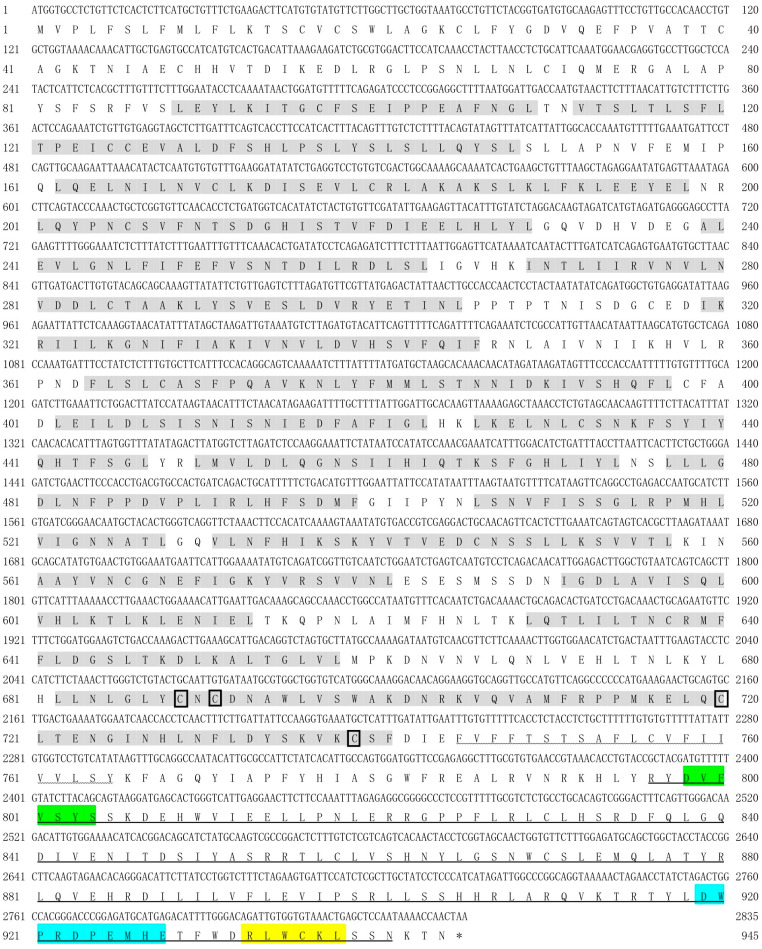
Nucleotide and deduced amino acid sequences and the domain architecture of *sp*TLR20. The shaded regions indicate the LRR domains, the wavy line marks the transmembrane domain, and the horizontal line represents the TIR domain. The cysteine residues marked within the boxes indicate conserved cysteine sites. The green region represents Box 1, the blue region represents Box 2, and the yellow region represents Box 3. * represents the stop codon.

**Figure 3:**
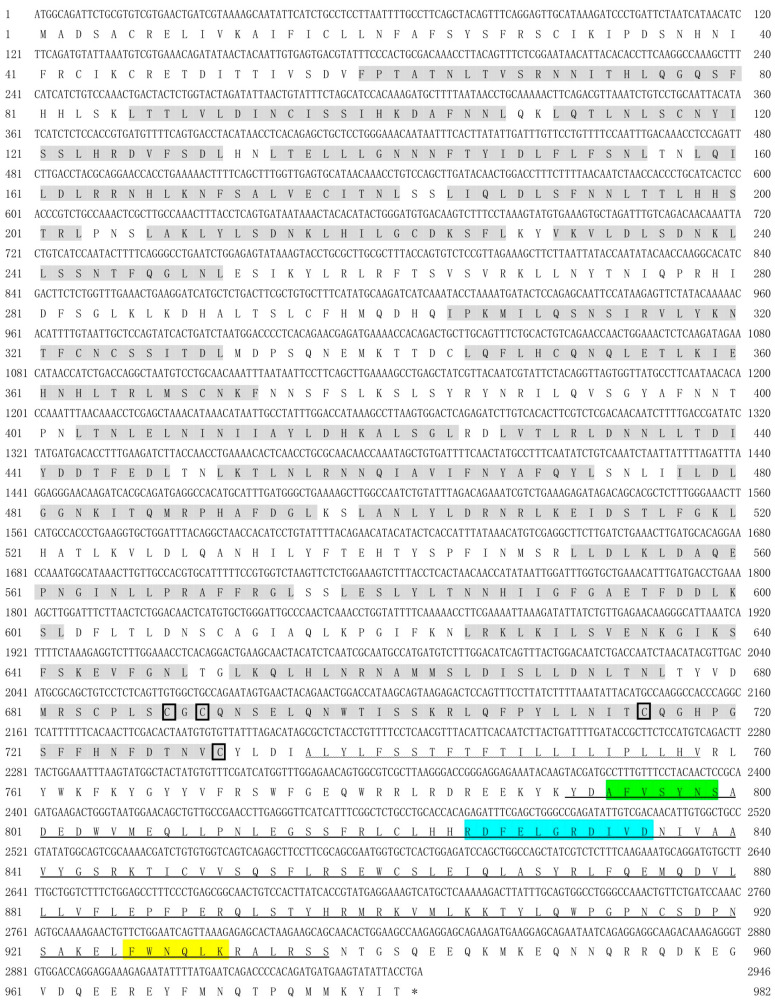
Nucleotide and deduced amino acid sequences and the domain architecture of *sp*TLR21. The shaded regions indicate the LRR domains, the wavy line marks the transmembrane domain, and the horizontal line represents the TIR domain. The cysteine residues marked within the boxes indicate conserved cysteine sites. The green region represents Box 1, the blue region represents Box 2, and the yellow region represents Box 3. * represents the stop codon.

**Figure 4:**
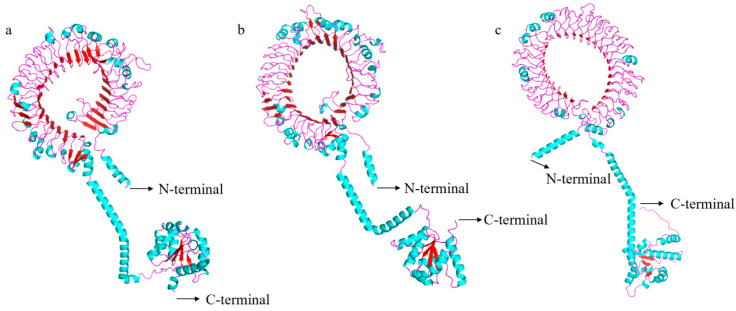
Predicted 3D structural models of the *sp*TLR19, *sp*TLR20, and *sp*TLR21 proteins. (**a**) 3D structure of predicted *sp*TLR19 protein. (**b**) 3D structure of predicted *sp*TLR20 protein. (**c**) 3D structure of predicted *sp*TLR21 protein. The blue regions indicate loop connections, the purple regions represent α-helices, and the red arrows denote β-strands.

The full-length coding sequences (CDS) of *spTLR19* were 2868 bp encoding a putative protein with 955 amino acids with a predicted molecular weight of 109.05 kDa and a theoretical pI of 5.97 (Figure 1). The full-length CDS of *spTLR20* was 2835 bp encoding a protein of 944 amino acids with a molecular weight of 107.81 kDa and a pI of 6.34 (Figure 2). While *spTLR21* possessed a 2946 bp CDS encoding a protein with 981 amino acids with a molecular weight of 113.85 kDa and a pI of 8.62 (Figure 3). Deduced amino acid sequences were analyzed, and the results indicated that the spTLRs cloned in this study possess the characteristics of TLR domain architecture comprising LRR domain, transmembrane domain, and TIR domain. The TIR domain included three conserved regions: Box 1, Box 2, and Box 3. The predicted 3D structures of three TLRs (spTLR19, spTLR20, and spTLR21) displayed a typical TLR conformation: the N-terminal LRR domain forming a horseshoe-like structure composed of multiple loops and β-strands, a central connecting region formed by α-helices, and a C-terminal globular region mainly composed of α-helices (Figure 4).

### 3.2. Alignment and Phylogenetic Analysis of spTLR19, spTLR20, and spTLR21

**Figure 5:**
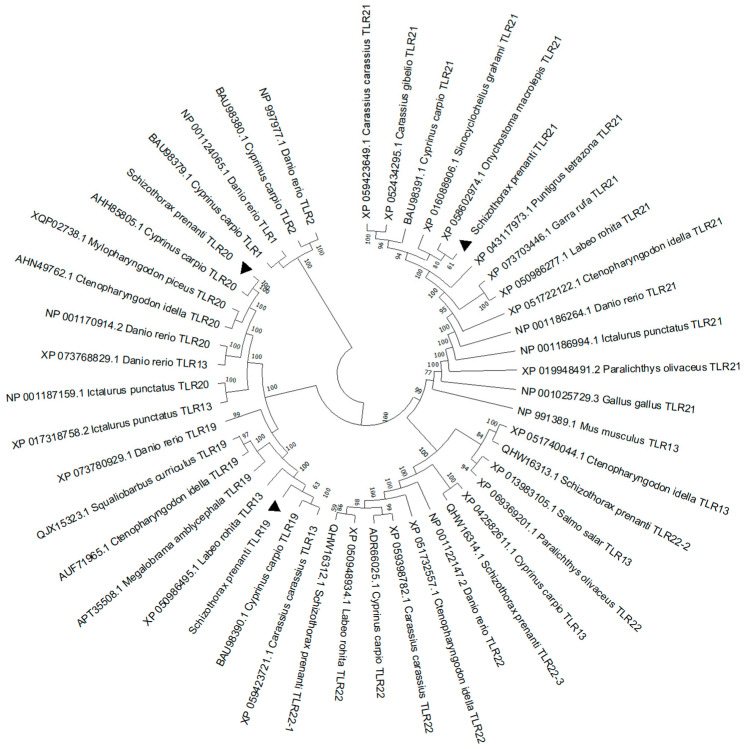
Phylogenetic tree illustrating the relationship between spTLR19, spTLR20, spTLR21, and other TLRs. The phylogenetic tree was generated by using the method of Neighbor-Joining in MEGA 11 software. 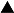 represents TLRs cloned in the present study.

To explore the evolutionary status of *sp*TLR19, *sp*TLR20, and *sp*TLR21, the phylogenetic tree was constructed based on full-length amino acid sequences. As shown in Figure 5, members of the TLR11 subfamily were clustered into a distinct clade, as were the members of the TLR1 subfamily. Among them, *sp*TLR19 and *sp*TLR20 were clustered more closely with *C. carpio* TLR19 (bootstrap values = 63) and TLR20 (bootstrap values = 100), respectively. Similarly, *sp*TLR21 was most closely related to *Onychostoma macrolepis* TLR21 (bootstrap values = 61).

The multiple sequence alignment indicated that three TLR sequences were highly conserved; furthermore, the TIR domain was more conserved compared to the LRR domain (Figures S1–S3). Homology analysis revealed high sequence similarity of the three TLRs with their homologs in other cyprinids. Specifically, *sp*TLR19 and *sp*TLR20 exhibited the highest amino acid identities to those in *C. carpio* (91.1% and 85.7%, respectively; Table 1 and Table 2), while *sp*TLR21 shares the highest identity with *O. macrolepis* (91.1%) TLR21 (Table 3).

### 3.3. Tissue Expression Patterns

**Figure 6:**
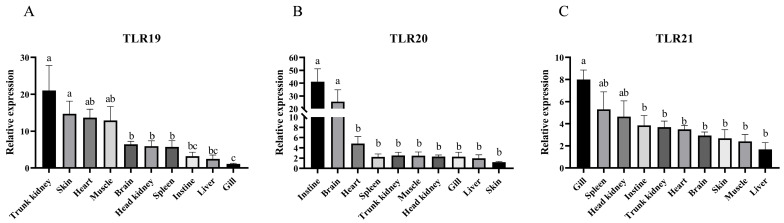
Tissue expression of *spTLR19*, *spTLR20*, and *spTLR21* in healthy *S. prenanti*. Relative expression levels of *spTLR19* (**A**), *spTLR20* (**B**), and *spTLR21* (**C**) in the head kidney, trunk kidney, brain, spleen, liver, gills, heart, muscle, intestine, and skin were analyzed using qRT-PCR, with 18S rRNA as the internal reference. Different lowercase letters (a, b, and c) indicate significant differences among the different tissues (*p* < 0.05). The expressions of *spTLR19* in gills, *spTLR20* in skin, and *spTLR21* in liver were chosen as calibrators, respectively. Data were shown as mean ± SEM (*n* = 3).

The tissue distribution analysis of *spTLRs* revealed that *spTLR19*, *spTLR20*, and *spTLR21* were ubiquitously expressed in ten examined tissues. *spTLR19* had the highest expression in the trunk kidney and skin, while its expression was minimal in the gills (Figure 6A). The expression of *spTLR20* was highest in the intestine and brain but was lowest in the skin (Figure 6B). *spTLR21* was most abundant in the gills and spleen, with the liver showing the lowest level (Figure 6C).

### 3.4. Expressions of spTLRs Following Bacterial Challenge

The expression patterns of *spTLR19*, *spTLR20*, and *spTLR21* were examined in the spleen, head kidney, liver, and gills following challenge with *S. agalactiae* and *A. veronii*, respectively.

**Figure 7:**
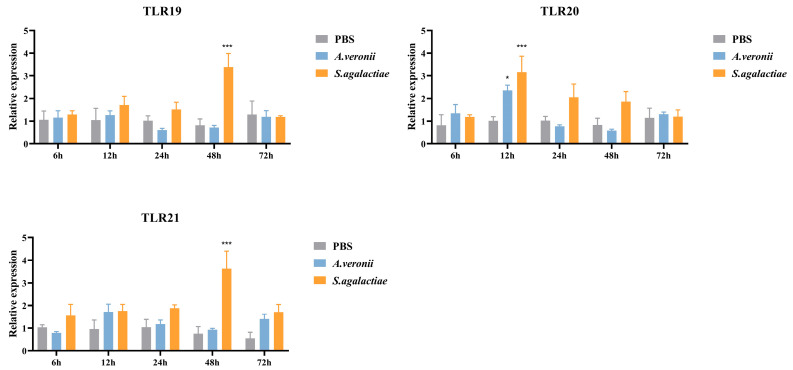
The relative expression levels of *spTLR19*, *spTLR20*, and *spTLR21* in spleen after stimulation with *S. agalactiae* and *A. veronii*. Expression levels of *spTLR19*, *spTLR20*, and *spTLR21* in the spleen were analyzed by qRT-PCR at 6 h, 12 h, 24 h, 48 h, and 72 h post-infection with *S. agalactiae* or *A. veronii*, using 18S rRNA as the internal reference. Statistical differences between the treatment groups and the control at each time point were indicated by asterisks (* *p* < 0.05, *** *p* < 0.001). The gene expression in the control group at corresponding time point was chosen as calibrator. Data were shown as mean ± SEM (*n* = 3), and the values were shown in Table S2.

In the spleen (Figure 7), stimulation with *S. agalactiae* significantly up-regulated the expression of *spTLR19* (3.39-fold, *p* < 0.001) and *spTLR21* (3.63-fold, *p* < 0.001) at 48 h post-infection (hpi), and *spTLR20* (3.17-fold, *p* < 0.001) at 12 hpi. In contrast, *A. veronii* challenge induced only a modest 2.36-fold (*p* < 0.05) increase in *spTLR20* expression at 12 hpi.

**Figure 8:**
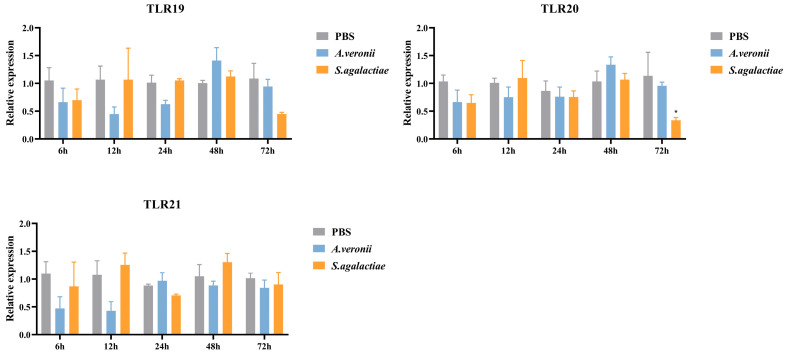
The relative expression levels of *spTLR19*, *spTLR20*, and *spTLR21* in head kidney after stimulation with *S. agalactiae* and *A. veronii*. Expression levels of *spTLR19*, *spTLR20*, and *spTLR21* in the head kidney were analyzed by qRT-PCR at 6 h, 12 h, 24 h, 48 h, and 72 h post-infection with *S. agalactiae* or *A. veronii*, using 18S rRNA as the internal reference. Statistical differences between the treatment groups and the control at each time point are indicated by asterisks (* *p* < 0.05). The gene expression in the control group at corresponding time point was chosen as calibrator. Data were shown as mean ± SEM (*n* = 3), and the values were shown in Table S2.

In the head kidney (Figure 8), *S. agalactiae* infection led to a decrease (0.34-fold, *p* < 0.05) in *spTLR20* expression at 72 hpi, while no significant changes were observed in the expressions of the three TLRs following *A. veronii* stimulation.

**Figure 9:**
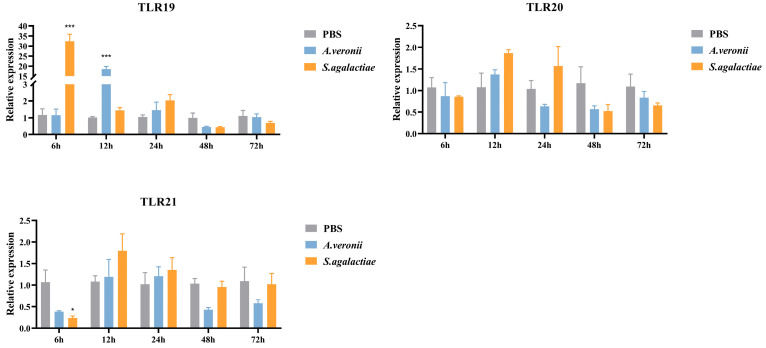
The relative expression levels of *spTLR19*, *spTLR20*, and *spTLR21* in liver after stimulation with *S. agalactiae* and *A. veronii*. Expression levels of *spTLR19*, *spTLR20*, and *spTLR21* in the liver were analyzed by qRT-PCR at 6 h, 12 h, 24 h, 48 h, and 72 h post-infection with *S. agalactiae* or *A. veronii*, using 18S rRNA as the internal reference. Statistical differences between the treatment groups and the control at each time point are indicated by asterisks (* *p* < 0.05, *** *p* < 0.001). The gene expression in the control group at corresponding time point was chosen as calibrator. Data were shown as mean ± SEM (*n* = 3), and the values were shown in Table S2.

In the liver (Figure 9), *S. agalactiae* infection induced a rapid and significant up-regulation (32.46-fold, *p* < 0.001) of *spTLR19* expression at 6 hpi, while *spTLR21* expression decreased (0.24-fold, *p* < 0.05) at the same time point. In contrast, challenge with *A. veronii* resulted in a marked increase (18.59-fold, *p* < 0.001) in *spTLR19* expression at 12 hpi, with no significant induction observed for *spTLR21*.

**Figure 10:**
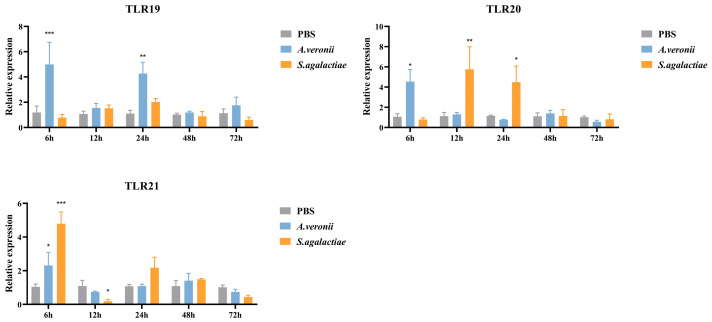
The relative expression levels of *spTLR19*, *spTLR20*, and *spTLR21* in gills after stimulation with *S. agalactiae* and *A. veronii*. Expression levels of *spTLR19*, *spTLR20*, and *spTLR21* in the gills were analyzed by qRT-PCR at 6 h, 12 h, 24 h, 48 h, and 72 h post-infection with *S. agalactiae* or *A. veronii*, using 18S rRNA as the internal reference. Statistical differences between the treatment groups and the control at each time point are indicated by asterisks (* *p* < 0.05, ** *p* < 0.01, and *** *p* < 0.001). The gene expression in the control group at corresponding time point was chosen as calibrator. Data were shown as mean ± SEM (*n* = 3), and the values were shown in Table S2.

In the gills (Figure 10), *S. agalactiae* infection induced distinct expression dynamics: a increase in *spTLR20* peaked at 12 hpi (5.75-fold, *p* < 0.01), before slightly subsiding to 4.49-fold (*p* < 0.05), while *spTLR21* expression peaked earlier at 6 hpi (4.79-fold, *p* < 0.001) and then declined significantly at 12 hpi (0.19-fold, *p* < 0.05). In contrast, *A. veronii* infection triggered up-regulation of *spTLR19* expression at 6 hpi (4.93-fold, *p* < 0.001) and 24 hpi (4.28-fold, *p* < 0.01), *spTLR20* at 6 hpi (4.54-fold, *p* < 0.05), and *spTLR21* at 6 hpi (2.31-fold, *p* < 0.05).

## 4. Discussion

In the TLR family, TLR19, TLR20, and TLR21 belong to the TLR11 subfamily [7], and phylogenetic analysis in the present study also supported this categorization. As a group of receptors with important functions, high diversity, and wide distribution in teleosts, the members of the TLR11 subfamily typically recognize proteins or nucleic acids [7,8]. TLR19 and TLR21 recruit adaptor TRIF (TIR domain-containing adaptor inducing IFN-β), then activate IFN and NF-κB and consequently induce the expression of type I interferon and inflammatory cytokines [7,12,19]. Although the ligand and adaptor were uncertain, TLR20 can activate the IFN and NF-κB signaling pathway and participates in antibacterial and antiviral immunity [7,16,17]. In this study, we respectively cloned *spTLR19*, *spTLR20*, and *spTLR21* from *S. prenanti* and analyzed their structural features. The complete CDS of *TLR19* (2868 bp), *TLR20* (2835 bp), and *TLR21* (2946 bp) were identified, all of these containing an LRR domain, a transmembrane domain, and a TIR domain. Furthermore, the close evolutionary relationship and high similarity of putative protein and structural domains indicated that *sp*TLR19, *sp*TLR20, and *sp*TLR21 may have similar functions with the corresponding homologues in other teleosts, respectively. Specifically, the LRR domain of *sp*TLR19 contains 21 LRR motifs, *sp*TLR20 consists of 18 LRR motifs, and *sp*TLR21 comprises 20 LRR motifs. However, previous studies have shown that the number of LRR motifs in TLR19, TLR20, and TLR21 varies among the different fish species [10,13,28,29]. For instance, the number of LRR motifs in *sp*TLR20 differs from those in *C. idella* and *C. carpio* TLR20 [30], and the LRR motifs in *sp*TLR21 are distinct from those in olive flounder (*Paralichthys olivaceus*) (19 LRR motifs) [31], rock bream (*Oplegnathus fasciatus*) (16 LRR motifs) [32], and large yellow croaker (*Larimichthys crocea*) (13 LRR motifs) [33]. Given that the LRR domain is responsible for binding ligands, whether the variation in LRR number among fish species changes the broad range capacity for recognition of different ligands of piscine TLRs remains to be elucidated.

In this study, *sp*TLR19, *sp*TLR20, and *sp*TLR21 conform to the conserved structural pattern. The typical 3D structure of TLRs consists of an extracellular region forming a characteristic horseshoe shaped the solenoid composed of multiple LRRs, usually located at the N-terminus and primarily responsible for ligand recognition, which determines TLR specificity [10,34,35]. In contrast, the TIR domain is located at the C-terminal cytoplasmic side, where it mediates interactions with downstream signaling pathways [36,37].

The tissue distribution patterns showed that most *spTLRs* were broadly expressed across all examined tissues, which is consistent with previous studies [38,39]. The wide expression indicated their potential role in the immune surveillance system in various tissues of the host. However, the tissue distribution patterns of these TLRs varied among the different fish species. For instance, *TLR19* exhibited higher expression in the brain, head kidney, and gills of *C. carpio* and in the head kidney and spleen of *P. fulvidraco* [13,29]. *TLR20* was more highly expressed in peripheral blood lymphocytes and intestine of *C. carpio* [30] and in the spleen, head kidney, and gills of Atlantic salmon (*Salmo salar*) [40]. Such variations may result from variation in fish species, development stages, and genetic background. Notably, the expression of *TLR21* appeared more conserved, with the highest levels consistently detected in the spleen and gills of *P. olivaceus* [31], *Labeo rohita* [41], *L. crocea* [33], and in the present study. Overall, these TLRs exhibited relatively higher expression levels not only in immune-related organs such as skin, intestine, gills, and spleen but also in other tissues, including brain, muscle, and trunk kidney, implying that they may have functions in addition to classical immune roles.

TLRs are indispensable components of innate immunity for the recognition of pathogens, and the members of the TLR11 subfamily could typically recognize nucleic acids and proteins [7,8]. After infection with *A. veronii*, TLR19 exhibited strong up-regulation, and this response pattern was also observed in *C. carpio* infected with *A. hydrophila* [29]. The ligand of TLR19 has been identified as double-stranded RNA (dsRNA) [12]. However, in this study, *spTLR19* showed immune responses to both *S. agalactiae* and *A. veronii*, and it was suggested that TLR19 could recognize additional ligands other than dsRNA. In addition, *spTLR19* showed a high response at early time points (6 and 12 hpi in the liver, 6 and 24 hpi in the gills). A similar early up-regulation was observed in the skin and intestine of common carp [29]. These findings imply that TLR19 may play an important role in early innate immune response within these tissues. The ligand for TLR20 remains undefined. Nevertheless, in this study, *spTLR20* was up-regulated by *A. veronii* infection. Moreover, a similar up-regulation of TLR20 following the *A. hydrophila* challenge has been reported in *C. idella* and *M. anguillicaudatus* [15,42], which was consistent with our research. Collectively, these results suggested that the components common to Gram-negative bacteria could be a potential ligand for TLR20. The TLR21 is known to recognize unmethylated CpG DNA [7]. In *O. fasciatus*, *TLR21* was also up-regulated in the spleen following *S. iniae* infection, peaking at 48 h [32], which was consistent with our findings. Interestingly, our results indicate that these TLRs exhibited a more pronounced response to *S. agalactiae*, a Gram-positive bacterium, compared to *A. veronii*, a Gram-negative bacterium. These results suggested that distinct recognition specificities of these receptors exist toward Gram-positive and Gram-negative pathogens. Nevertheless, the precise ligands responsible for triggering these responses remain to be identified, and further studies are required to elucidate their ligand-binding mechanisms.

## 5. Conclusions

In conclusion, *spTLR19*, *spTLR20*, and *spTLR21*, belonging to the TLR11 subfamily, were identified in this study. Sequence and phylogenetic analyses confirmed that these three receptors are relatively conserved among the teleosts. *sp*TLR19 and *sp*TLR20 were clustered most closely with the TLR19 and TLR20 of *C. carpio*, while *sp*TLR21 was grouped with the TLR21 of *O. macrolepis*. Tissue distribution showed that these *spTLRs* were relatively high expression in immune-related organs such as the spleen and gills, as well as in the skin and intestine. Following infection with *S. agalactiae* and *A. veronii*, three TLRs exhibited marked up-regulation, and it was suggested that their potential roles in antibacterial immune responses. Collectively, these findings enhance our understanding of the functional roles of TLRs in antibacterial defense and provide a theoretical basis for future studies on innate immunity, with potential implications for disease prevention and control in aquaculture.

## Figures and Tables

**Table 1:** Homology analysis of *sp*TLR19.

		Identity%					
		*S. prenanti*	*C. carpio*	*D. rerio*	*C. idella*	*M. amblycephala*	*S. curriculus*
Similarity%	*S. prenanti*		91.1	71.5	81.5	77.2	82.3
	*C. carpio*	91.3		70.5	80.0	76.4	80.2
	*D. rerio*	73.7	72.5		71.9	67.5	71.3
	*C. idella*	82.4	80.7	74.0		86.8	94.0
	*M. amblycephala*	81.1	80.1	72.8	91.0		86.5
	*S. curriculus*	83.2	80.9	73.4	94.0	90.7	

Note: The homology analysis performed using BioEdit 7.2.5.0. Same to Table 2 and Table 3.

**Table 2:** Homology analysis of spTLR20.

		Identity%					
		*S. prenanti*	*M. piceus*	*D. rerio*	*C. carpio*	*C. idella*	*P. tetrazona*
Similarity%	*S. prenanti*		77.9	68.2	85.7	78.2	84.5
	*M. piceus*	78.3		69.2	76.9	90.8	74.5
	*D. rerio*	69.5	70.4		67.6	69.0	66.8
	*C. carpio*	86.1	77.1	68.8		77.8	80.2
	*C. idella*	78.8	91.0	70.2	78.2		74.5
	*P. tetrazona*	84.9	74.7	68.0	80.2	74.9	

**Table 3:** Homology analysis of spTLR21.

		Identity%					
		*S. prenanti*	*O. macrolepis*	*C. carpio*	*C. gibelio*	*C. carassius*	*D. rerio*
Similarity%	*S. prenanti*		91.1	90.9	88.3	89.6	77.1
	*O. macrolepis*	92.2		92.3	89.5	90.2	78.8
	*C. carpio*	91.4	93.1		92.5	92.8	78.6
	*C. gibelio*	88.9	90.4	92.7		94.3	76.9
	*C. carassius*	90.3	91.2	93.1	94.7		77.5
	*D. rerio*	78.0	79.9	79.4	77.8	78.5	

## Data Availability

The original contributions presented in this study are included in the article/Supplementary Material. Further inquiries can be directed to the corresponding author(s).

## Supplementary Materials

The following supporting information can be downloaded at: https://www.mdpi.com/article/10.3390/ani16030511/s1, Table S1: primers for cloning and qRT-PCR; Table S2: the mean ± SEM values of spTLRs expression levels following bacterial challenge; Figure S1: multiple sequence alignment of TLR19; Figure S2: multiple sequence alignment of TLR20; Figure S3: multiple alignments of TLR21.
