# Supplementary material for: Molecular Cloning and Expression Responses to Streptococcus agalactiae and Aeromonas veronii of TLR19, TLR20, and TLR21 in Schizothorax prenanti
Source: Animals (Basel). 2026 Feb 5;16(3):511. doi: 10.3390/ani16030511 (PMC12897282; doi:10.3390/ani16030511)
Supplement: Supplementary file 1 [file animals-16-00511-s001.zip › Figure S2.docx]

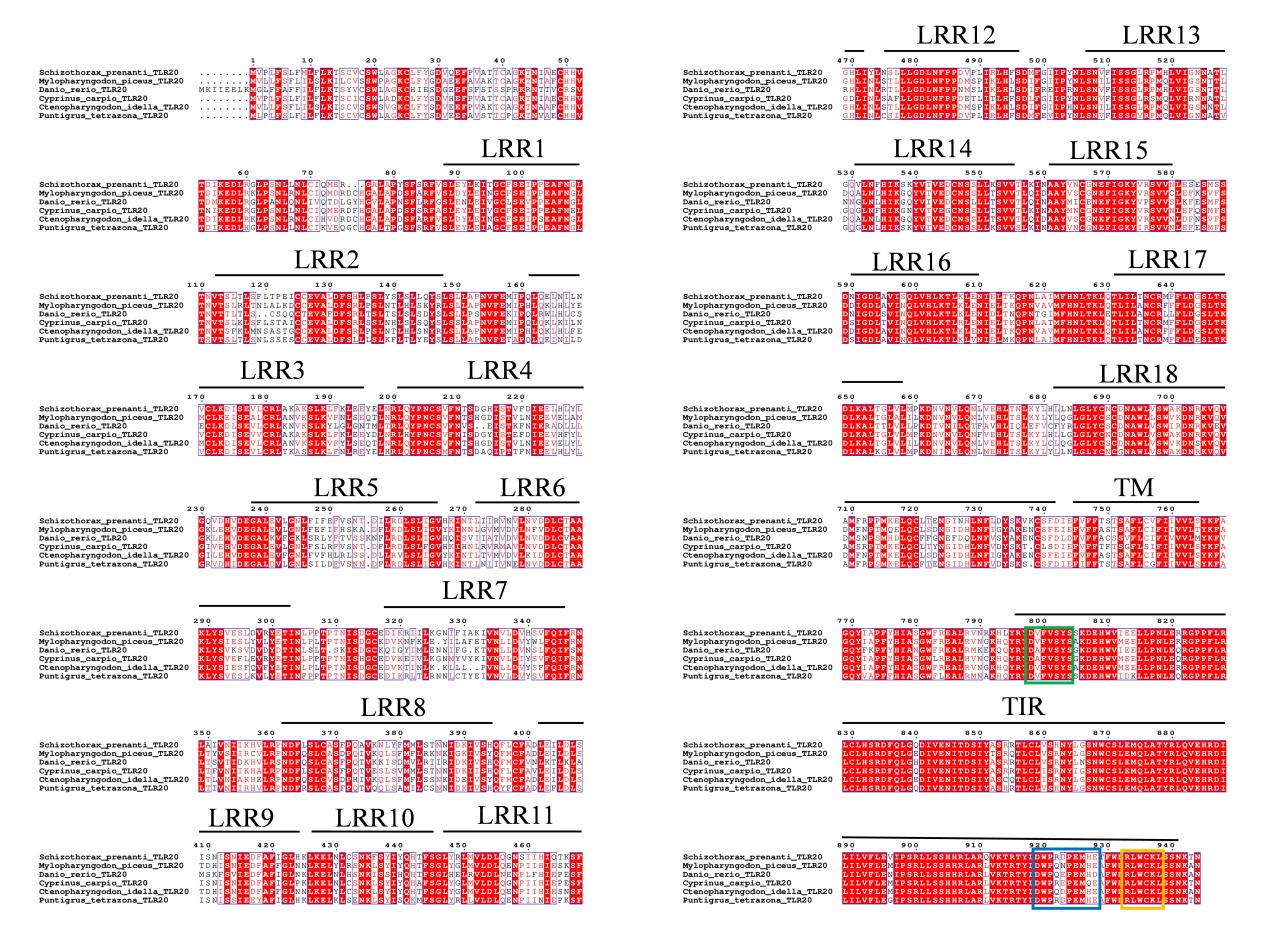


**Figure S2.** Multiple sequences alignment of TLR20. Mutiple sequences alignment was carried out using the software MEGA 11. Species names (GenBank accession number) were listed as *Mylopharyngodon piceus* (XQP02738.1), *D. rerio* (NP_001170914.2), *C. carpio* (AHH85805.1), *C. idella* (AHN49762.1), and *Puntigrus tetrazona* (XP_043105158.1). Regions marked by green, blue, and yellow boxes correspond to Box1, Box2, and Box3, respectively.
