# Supplementary material for: Molecular Cloning and Expression Responses to Streptococcus agalactiae and Aeromonas veronii of TLR19, TLR20, and TLR21 in Schizothorax prenanti
Source: Animals (Basel). 2026 Feb 5;16(3):511. doi: 10.3390/ani16030511 (PMC12897282; doi:10.3390/ani16030511)
Supplement: Supplementary file 1 [file animals-16-00511-s001.zip › Table S1.docx]

**Table S1.** Primers for cloning and qRT-PCR.

| **Primers** | **Sequences(5’-3’)** |
| --- | --- |
| Primers for CDS cloning | |
| TLR19 F | ATGGGTGTGCATGACTCCA |
| TLR19 R | TCAAGAAGCTTCCATGTCTTCCT |
| TLR20 F | ATGGTGCCTCTGTTCTCAC |
| TLR20 R | TTAGTTGGTTTTATTGGAGCTCAG |
| TLR21 F | ATGGCAGATTCTGCGTGTC |
| TLR21 R | TCAGGTAATATACTTCATCATCTGTG |
| Primers for qRT-PCR | |
| TLR19 F | ATTGCGTTTCAGAACCTCT |
| TLR19 R | TATCCAGTGACTCGCCTAT |
| TLR20 F | ATAATGCGTGGCTGGTGTC |
| TLR20 R | CTGGCAATGTGATAGAATGG |
| TLR21 F | TTTGATACCGCTTCTCCAT |
| TLR21 R | GTTCCATTACCCAGTCTTC |
| 18S rRNA F | ACCACCCACAGAATCGAGAAA |
| 18S rRNA R | GCCTGCGGCTTAATTTGACT |
