# Supplementary material for: Molecular Cloning and Expression Responses to Streptococcus agalactiae and Aeromonas veronii of TLR19, TLR20, and TLR21 in Schizothorax prenanti
Source: Animals (Basel). 2026 Feb 5;16(3):511. doi: 10.3390/ani16030511 (PMC12897282; doi:10.3390/ani16030511)
Supplement: Supplementary file 1 [file animals-16-00511-s001.zip › Figure S1.pdf]

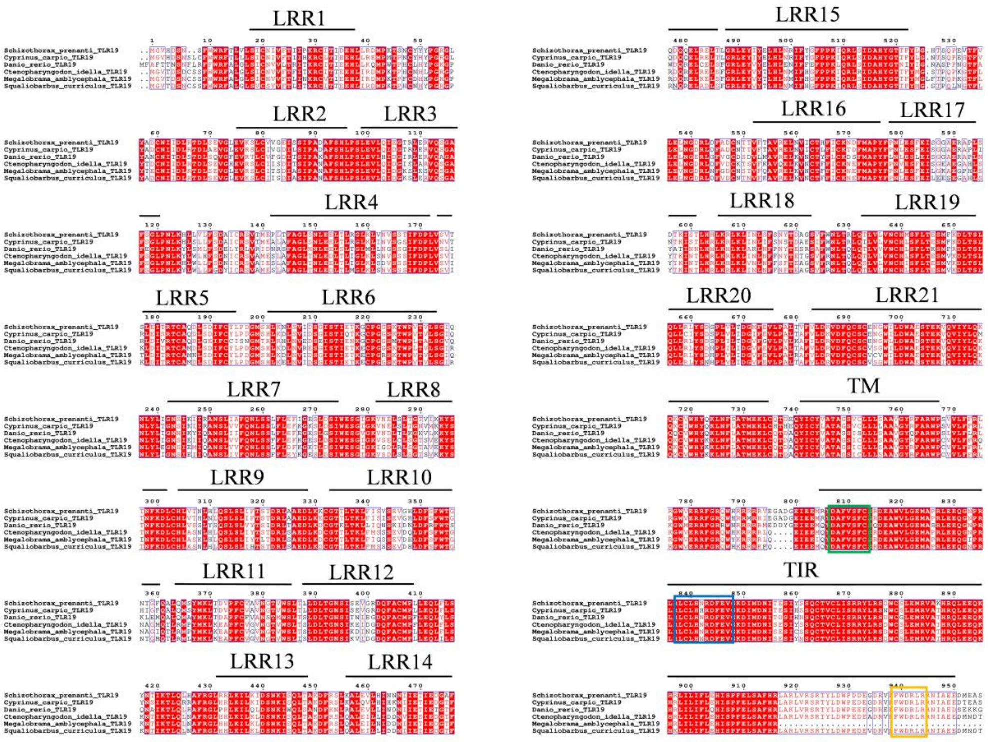

Figure S1. Multiple sequences alignment of TLR19. Mutiple sequences alignment was carried out using the software MEGA 11. Species names (GenBank accession number) were listed as *C. carpio* (BAU98390.1), *D. rerio* (XP_073780929.1), *Ctenopharyngodon idella* (KY798320.1), *Megalobrama amblycephala* (APT35508.1) and *Squaliobarbus curriculus* (QJX15323.1). Regions marked by green, blue, and yellow boxes correspond to Box1, Box2, and Box3, respectively.
