# Supplementary material for: Molecular Cloning and Expression Responses to Streptococcus agalactiae and Aeromonas veronii of TLR19, TLR20, and TLR21 in Schizothorax prenanti
Source: Animals (Basel). 2026 Feb 5;16(3):511. doi: 10.3390/ani16030511 (PMC12897282; doi:10.3390/ani16030511)
Supplement: Supplementary file 1 [file animals-16-00511-s001.zip › Figure S3.pdf]

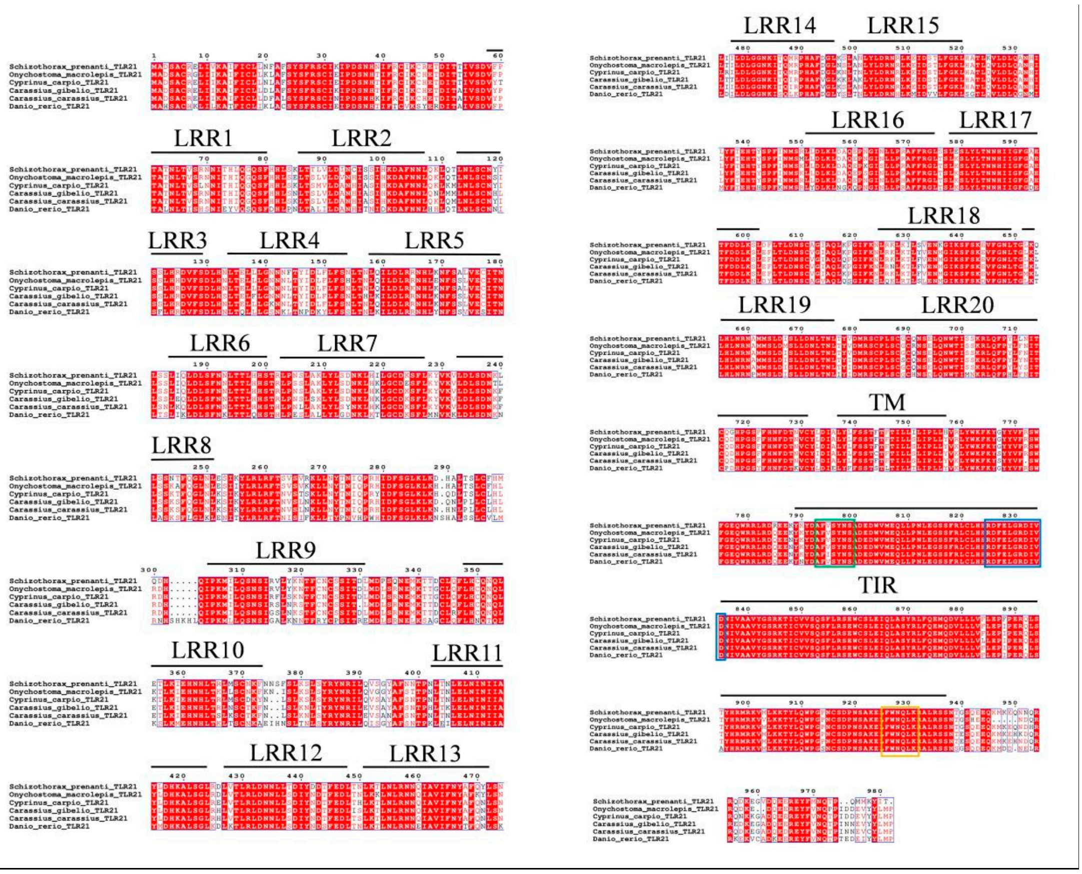

Figure S3. Mutiple sequences alignment of TLR21. Mutiple sequences alignment was carried out using the software MEGA 11. Species names (GenBank accession number) were listed as *O. macrolepis* (XP_058602974.1), *C. carpio* (BAU98391.1), *Carassius gibelio* (XP_052434295.1), *Carassius carassius* (XP_059423649.1), and *D. rerio* (NP_001186264.1). Regions marked by green, blue, and yellow boxes correspond to Box1, Box2, and Box3, respectively.
