# Supplementary material for: Molecular Cloning and Expression Responses to Streptococcus agalactiae and Aeromonas veronii of TLR19, TLR20, and TLR21 in Schizothorax prenanti
Source: Animals (Basel). 2026 Feb 5;16(3):511. doi: 10.3390/ani16030511 (PMC12897282; doi:10.3390/ani16030511)
Supplement: Supplementary file 1 [file animals-16-00511-s001.zip › Table S1.pdf]

Table S1. Primers for cloning and qRT-PCR.

Primers	Sequences(5′-3′)
Primers for CDS cloning	
TLR19 F	ATGGGTGTGCATGACTCCA
TLR19 R	TCAAGAAGCTTCCATGTCTTCCT
TLR20 F	ATGGTGCCTCTGTTCTCAC
TLR20 R	TTAGTTGGTTTTATTGGAGCTCAG
TLR21 F	ATGGCAGATTCTGCGTGTC
TLR21 R	TCAGGTAATATACTTCATCATCTGTG
Primers for qRT-PCR	
TLR19 F	ATTGCGTTTCAGAACCTCT
TLR19 R	TATCCAGTGACTCGCCTAT
TLR20 F	ATAATGCGTGGCTGGTGTC
TLR20 R	CTGGCAATGTGATAGAATGG
TLR21 F	TTTGATACCGCTTCTCCAT
TLR21 R	GTTCCATTACCCAGTCTTC
18S rRNA F	ACCACCCACAGAATCGAGAAA
18S rRNA R	GCCTGCGGCTTAATTTGACT
